# Memory-Modulation: Self-Improvement or Self-Depletion?

**DOI:** 10.3389/fpsyg.2018.00469

**Published:** 2018-04-05

**Authors:** Andrea Lavazza

**Affiliations:** Neuroethics, Centro Universitario Internazionale, Arezzo, Italy

**Keywords:** memory-erasing, propanolol, autobiographical memory, regulative conceptions of self, rigid identity, extended identity

## Abstract

Autobiographical memory is fundamental to the process of self-construction. Therefore, the possibility of modifying autobiographical memories, in particular with memory-modulation and memory-erasing, is a very important topic both from the theoretical and from the practical point of view. The aim of this paper is to illustrate the state of the art of some of the most promising areas of memory-modulation and memory-erasing, considering how they can affect the self and the overall balance of the “self and autobiographical memory” system. Indeed, different conceptualizations of the self and of personal identity in relation to autobiographical memory are what makes memory-modulation and memory-erasing more or less desirable. Because of the current limitations (both practical and ethical) to interventions on memory, I can only sketch some hypotheses. However, it can be argued that the choice to mitigate painful memories (or edit memories for other reasons) is somehow problematic, from an ethical point of view, according to some of the theories of the self and personal identity in relation to autobiographical memory, in particular for the so-called narrative theories of personal identity, chosen here as the main case of study. Other conceptualizations of the “self and autobiographical memory” system, namely the constructivist theories, do not have this sort of critical concerns. However, many theories rely on normative (and not empirical) conceptions of the self: for them, the actions aimed at mitigating or removing specific (negative) memories can be seen either as an improvement or as a depletion or impairment of the self.

## Forgetting on demand, the self, and the autobiographical memory

The human being has always tried to have control over his memory. In ancient times, when there were no external media to preserve data in an easily accessible manner, what today we call “declarative memory” was crucial for scholars or those exercising intellectual professions. The enhancement of that type of memory was sought with techniques like the *loci*, namely the association of information to well-known places and objects (Yates, 1966). But already then it was clear that memory was not a mere instrument, regardless of how it is used and accessed. Plato, for example, questioned writing as a way to preserve thought: in *Phaedrus*, Socrates says that writing is fixed and therefore “encloses” the contents of the message.

Autobiographical memory, on the other hand, has always been ambivalent: usually people want it to be precise and always available, but it can also be a curse. Remembering events and feelings of our lives, for many philosophers and most people, is conceived of as the essence or basis of personal identity. So, diseases such as Alzheimer's, which damage memory, are considered one of the worst possible tragedies. On the other hand, being able to forget unpleasant facts and negative emotions would in many cases appear to be a liberation, enabling one to live better without the weight of painful or disturbing memories.

Even the *Odyssey*, one of the founding works of Western culture, mentions the idea of oblivion through pharmacological interventions. Classical writers such as Cicero and Petrarch narrate that the Athenian leader Themistocles, having learnt the art of remembering taught by Simonides, said that he rather would have learnt the art of forgetting. *Ars oblivionalis* has always been an unreachable goal and yet it has been evoked and desired throughout the centuries.

On the one hand, we are aware—and contemporary psychological and neuroscientific research confirm this—that there is a constant process of acquisition of sensory data and information in general, most of which, despite passing through the filter of conscious attention, is soon forgotten (Baddeley et al., 2014). This is due to a principle of energetic economy embedded in the evolution of our body, but it is also an unconscious selection that relieves us of a mnemonic load that would be otherwise unbearable, both cognitively and emotionally. Consider the difficulties experienced by those who suffer—the term is appropriate both in the clinical and in the existential sense—from hypermnesia (Ally et al., 2013).

On the other hand, the drastic mnemonic selection of incoming data, the progressive loss of precision of memory over time and the fatigue entailed by storing a complete set of new data often make us wish for a larger and better accessible memory (supposing that the failed recollection of memories is due to an access problem rather than to the loss of the mnemonic trace). At the same time, the persistence of some memories clashes with our will to not have them in our consciousness. Hence the paradox by which the more you seek to ignore a given object or event, the more it comes to consciousness, alone or in association with other thoughts (Wegner et al., 1987).

The fact that an effective psychological forgetting technique is almost impossible, despite the attempts made, has discouraged neither the desire to forget nor the research on the cerebral bases of memory. The hypotheses of chemically-induced oblivion have long been a matter of science fiction—except for the massive memory damage caused by the unwanted side-effects of alcohol, psychoactive substances, and electroconvulsive therapy—so the reflection on the consequences of this “forgetfulness on demand” has not been very specific. However, recently, several neuroethicists have addressed the use of new possible tools to erase or mitigate negative personal memories (President's Council on Bioethics, 2003; Liao and Sandberg, 2008; Lavazza, 2015).

It is well-known that a huge amount of human and financial resources is being invested in trying to block memory loss caused by neurodegenerative diseases. And there is an ongoing ethical debate on the condition of those who have lost all or part of their autobiographical memories. In this condition, is one still a “person” and should one be treated as such? How much autonomy can one have? Does one still have authentic preferences and wishes? Now, consider new tools to modify or adjust memories, provided they are ever available: would they lead to a similar set of questions? Or would this be a different level of philosophical, neuroethical and psychological investigation compared to the one that focuses on the consequences of Alzheimer's? (Dworkin, 1986; Dresser, 1992; Jaworska, 1999; Strohminger and Nichols, 2015).

The common idea underlying these considerations is that autobiographical memory is strongly linked to the self and the identity of the individual, and this view is neither new nor original. However, in this paper I want to introduce an interpretative framework linked to the recent discovery of molecules that seem to be able, for the first time, to effectively modulate autobiographical memory by reducing the emotional reach of salient, negative and painful memories, according to the studies that have been carried out so far. The opportunity to intervene on memory “by subtraction” raises questions and perplexities. This seems to be due to the fact that we have certain ideas of the self and of personal identity, as well as of their relationship with memory.

The first point I would like to address is that attempts to give an axiological evaluation of memory modulation-removal interventions are based on conceptions and models of personal identity and the self that act as reference points also for a wider set of values. The ethical discussion on the chemical modulation of memories can thus be helpful to clarify and assess the relationship between self and autobiographical memory especially from a normative point of view (Selimbegović et al., 2016).

But there is a second point that seems to deserve greater attention, because perhaps it has not been sufficiently emphasized in the literature. Some conceptions and models of the self are based on empirical research, and others are essentially normative: that is, they are the direct result of scholarly reflection or the result of social and cultural processes due to the convergence of various elements. All of these conceptions and models of the self work as references for the evaluation of autobiographical memory interventions. So, potential judgments on memory modulation-removal can show that the relationship between self and autobiographical memory is extremely complex. In fact, there are competing empirical models and also normative concepts that have a significant persistence and play an important role in guiding both judgment and behavior. In this paper, I especially focus on narrative theories of personal identity and related normative issues about memory-modulation.

## How to modulate memories

As has been said, it is only recently that external tools have shown the potential to modulate memory in living things, specifically to modify memories. This is not the place to describe the specific action modes of the various techniques tested. In animal models, it was possible to achieve the deletion of specific mnestic traits (at least in their behavioral manifestation) and to modify the salience of individual memories (evaluated by the search for or avoidance of behaviors that they arouse).

In human beings, because of the centrality and importance that memory has for the individual, it has of course been more complicated to make experiments. The most promising way, in the current state of research, seems to be related to molecules able, at least in many cases, to attenuate the emotional reach of autobiographical memories, making them less salient to the subject. I will not enter into the detail of the neuroscientific and clinical debate on the safety and efficacy of the treatment, since the focus of the paper is what the possible availability of such a treatment, presumably considered effective, can tell us about the relationship between autobiographical memory, self and personal identity. In this sense, it is useful to start with a definition.

(Def1) “Memory-editing is a psychological (modification of the associative processes related to memories) or neurobiological (pharmacological and/or optogenetic) intervention in order to weaken or change the subjective negative valence of autobiographical memories or completely remove the memory trace of an autobiographical event” (Lavazza, 2017).

The memory-editing technique to which I will refer is that based on the administration of a particular molecule in certain temporal windows related to the memory on which it is intended to act: propranolol. As noted by James McGaugh, this molecule—normally used as a beta-blocker in hypertension treatments—can be helpful in diminishing the emotional effect of a given memory (Cahill et al., 1994). Situations of emotional turmoil are normally connected to the production of adrenaline and cortisol (the so-called “stress hormones”), which in turn lead to a significant increase in the neurotransmitter norepinephrine. The latter has a dual function: it connects the given memories to the fear circuit and causes anxiety, with symptoms such as tachycardia, tremor, or sweating. β-adrenergic receptors of the basolateral amygdala (β1 and β2), which norepinephrine binds to, produce a stream of molecules signaling the codification of the memory to the brain. This is a simplified description, shared by only some scientists. However, according to this view, propranolol blocks the activation of the receptors, thus preventing the (re)consolidation of a given memory.

According to early research, there is a connection between stress hormones and declarative memory: if an event causes emotional arousal, it is likely to produce a stronger and longer-lasting memory (Pitman et al., 2002). Propranolol only acts in such cases, if taken during or shortly after the stressful event, but is inefficacious on emotionally “bland” memories. This shows that if the adrenaline and noradrenaline produced by the adrenal glands are inhibited, as a consequence, the mnemonic process loses its emotional component: the subject thus preserves his or her declarative memory *without* the strong (negative) emotional component that would otherwise come with it, affecting given vital signs (respiration, heart rate, blood pressure) and arousing feelings of distress, fear or anxiety.

According to several findings related to experiments with propranolol, the molecule appears to work if taken shortly after the emotionally stressful event. For examples, victims of car accidents have shown to have less intense memories of the traumatic event if they take propranolol, which is the more efficacious the earlier it is taken (Brunet et al., 2011). Also, subjects suffering from post-traumatic stress disorder (PTSD) generally showed no benefit from the molecule (Muravieva and Alberini, 2010). Nevertheless, on healthy subject propranolol is known to be effective in weakening fear responses (Kindt et al., 2009), more than what usually happens according to Pavlov's paradigm.

To make one example, subjects suffering from arachnophobia have been able to overcome it by weakening the sensation of fear produced by seeing a spider, despite being still conscious of the potential danger it may cause. In addition to working on the immediate fear response, as previously mentioned, propranolol can affect the subsequent memories of a stressful event. This can happen both if taken shortly after the event, and by working on the reconsolidation of old memories. Reconsolidation is a mechanism by which recalling a memory activates a complex molecular process in the subject's nervous system, as a result of which the memory is biologically malleable (Nader et al., 2000; Nader and Hardt, 2009). This is the reason why often recalled episodes slowly change in time. Based on the fact that reconsolidation makes memories malleable, propranolol can be used to weaken traumatic memories even some time after the stressful event (Brunet et al., 2011; Soeter and Kindt, 2011; Elsey and Kindt, 2016).

Therefore, if taken during the recollection of a stressful event, propranolol will weaken the emotional component of the memory attached to it, while leaving the declarative memory of it untouched. For example, the subject may still remember being attacked one night when coming back home, but without the negative emotional burden and the related activation of the autonomic system, which can lead up to PTSD. Propranolol is effective for a short time period (a few hours) following the traumatic event and acts in a preventive way if taken during that period of time. When the traumatic event has already fixated in memory, one can try to intervene by explicitly recalling the memory in question while taking propranolol: in this case, the latter acts by exploiting the malleability of memory in the reconsolidation phase.

The effect of the intake of propranolol—immediately after the traumatic event or while explicitly recalling it to consciousness, when the biochemical processes lead to its cerebral reconsolidation—is that the memory will probably become less painful, if not emotionally neutral. As a consequence, it will be less able to motivate the subject by influencing her preferences, intentions and choices. This aspect is what I want to address now.

## What is wrong with “deleting” a memory?

Since it is possible to intervene on memories, the question has arisen whether to act on memory is ethically permissible or recommendable. An example that can help tackle this point was offered by Erler (2011). There are two friends, Elisabeth and Sonya, who have both been bullied in high school. Despite this, they managed to finish school and lead a rather satisfactory life. However, later on Elisabeth starts experiencing the consequences of her past: when the former classmates invite the girls to parties and various activities, Sonya joins in with full ease and peace of mind, whereas Elisabeth cannot. She demands an apology first, as she thinks they have acted immorally, in a way that has scarred her deeply. On the other hand, Elisabeth wishes she were like Sonya, so that she could forget the past and not be stuck in painful memories. So she chooses to take propranolol: the negative memories of school fade, their emotional component almost disappears. Elisabeth remembers the facts, but with no pain or resentment. Now she can see her former classmates as if she had truly forgiven them, even though they never said sorry. Her life seems happier and more carefree: she no longer thinks of bullism, and her wellbeing has apparently increased.

However, the question arises whether Elisabeth's choice was authentic, in line with her “true” self. Of course, the very idea of a “true self” can be questioned, but it is undeniable that people have personality traits (partly genetic) and long-term personal preferences and aversions (such as sexual orientation, a given lifestyle, a religious belief etc.). These characteristics make up (at least partly) a person's identity or self, guiding him in his behavior and in his reactions. If a long-term choice (say, veganism) is perceived as defining, we often publicly declare it and stay loyal to it even when the given situation might induce us to make an exception (say, an important business meeting where they only serve meat).

In this sense, autobiographical memory has to be coherent, functioning as a tool to limit or guide the fundamental orientations underlying the self. According to Erler (2011), the chemical alteration of one's memory might lead to non-authentic choices. In Elisabeth's case, for example, she ends up “forgiving” her schoolmates without them apologizing, as would have been the demand of her “true” self. Indeed, Elisabeth's convictions—her *self* —would not have allowed her to make this choice, as it would have contradicted her general beliefs. The modification of part of her autobiographical memory, especially the emotional salience of some events, has made it so that the latter lost the motivating power they had before.

It could be objected that someone with good cognitive skills and a solid moral orientation would still judge an evil deed negatively. This would probably hold true for—say—rape, but bullism is a set of many little acts that, taken one by one, may be considered relatively harmless “pranks.” Therefore, it is likely that bullism would arouse retrospective pain in the victim only if accompanied by the negative emotional component of its memories. A well-functioning autobiographical memory would be needed to this effect. So does it mean that Elisabeth “betrayed” her true self? And what about Sonya? Perhaps she doesn't share Elisabeth's moral inclination, or perhaps her memories are “naturally” less strong and distressing. Or else, she finds it easier to move on and leave the past behind.

Either way, it seems that without propranolol Elisabeth would not have forgiven her classmates without them acknowledging their wrongdoing. Also, her morality drives her to fight bullism publicly precisely because she has undergone it herself. But if the motivational aspect of the memory fades, her commitment might also become weaker (victims of abuse or discrimination are often the best candidates to fight such things). However, one cannot overlook a possible objection to Erler's argument, namely that the equivalence between the act of forgiving, which is a process and an experience with a relevant social dimension, and the individual experience of forgetting is far from obvious. If there may be a cause-effect link between the assumption of propranolol and “forgiveness,” it is still true that forgiveness, as it is generally understood, is a conscious process that takes into consideration the fact in question and elaborates it consciously, overcoming it in relation to its interpersonal effects without forgetting it on a personal level.

In this sense, it is important to recall the already mentioned concept of authenticity. It might be defined as follows:

(Def2) “Authenticity is the consistency (and the second order identification of one's own desires, *a la* Frankfurt) of the choices made by the individual—obvious choices or ones with potentially observable effects—with the individual's identity (at any given time), or at least with some of the relevant identity components for the choice in question” (Lavazza, 2017).

This concept is based on what may be called *rigid identity*, that is, something given, tied to a self that tends to remain stable over time. This definition incorporates a normative component: not only is the self-stable, but it should be maintained such. Of course, the definition that I propose cannot be exhaustive of all theories of authenticity throughout history. In this sense, one should also consider the existential-phenomenological literature, which at least partially originated, especially with Heidegger (1927-1962), the very concept of authenticity. For Jaspers (1919), authenticity is what is most profound as opposed to what is more superficial; for example, what affects the core of every psychic existence as opposed to what only touches the surface, what lasts as opposed what is momentary, what has grown and developed with the person as opposed to what the person has accepted or imitated. In the same vein, Heidegger and Sartre have a “strong” conception of authenticity not as something true to a pre-given self but as fidelity to the true self. It should be understood as a construction that takes place in a relationship, and this relationship has the purpose of a whole life project which, from time to time, incorporates elements of the present situation. And this ideal of authenticity is connected to being a person of a particular sort with the virtues of integrity and perseverance. In a “weak” form this view can be connected to the contemporary theories of identity as a narrative rather than to the idea of rigid identity, which however recalls a widespread idea of authenticity as coherence with something given in the person.

(Def3) The notion of *rigid identity* in the modern age originates from the idea of an “original entity” in the metaphysical sense (following Descartes) which was later considered to be of a psychological nature (following Locke), but dates back to ancient and widespread intuitions and concepts developed in many cultures; it is conceived of as the self-consciousness of a thinking self, rather than of an extended body—an identity core of the subject that has ideal more than real value. This identity may partly change over time, but always maintaining a stable core, namely something that characterizes the person and that can be discovered by different means, because it is sometimes concealed by external influences. It is what we think makes us unique and therefore must be preserved and not sacrificed.

The notion of rigid identity is an extremization and an idealization of the unsophisticated intuition of “core self,” which is not a metaphysical substance, but a persistent set of consistent psychological traits and features (whose origin may be genetic or due to parental and environmental influences during childhood). There is evidence supporting this notion both from the empirical (Klein, 2013) and from the philosophical standpoint. Consider, for example, Rawls' theory of justice. Rawls presupposes an *original position* in which, thanks to a “veil of ignorance,” the subjects are unaware of their personal characteristics and of the sociohistorical context. The implication is that, even without a rich biography, human beings preserve a (non-lockean) identity by which to make meaningful decisions in a consistent way.

Another example is that described by Damasio of a severely amnesic patient, David, who had an autobiographical memory span of less than a minute (Damasio, 1999. chs. 2, 4). In this case, we are faced with a person who, due to an encephalitis, can neither live in the past nor project himself consistently in the future, nor evaluate the consequences of his actions. This individual therefore does not have a personal identity in the sense of psychological continuity, according to the classic Lockean criterion^1^. Yet, based on the description of his behavior, we can infer that his present self and his short-term goals are vital and adequately related to reality. Moreover, thanks to mnestic traces present at an implicit level of which he is not conscious, David manages to be coherent with himself, with his tastes and behavioral choices. And for this reason, thanks to this minimal core of rigid identity, he can be fully considered a person, even if he has lost the explicit mnemonic continuity that for many constitutes the basic criterion of personal identity (cf. Meini, 2017).

Some claims that personal identity is not logically presupposed by memory (cf. Bernecker, 2010) and hence they can deny that there are circularity objections to accounts of personal identity based on memory. But it is likewise argued that memory presuppose personal identity (Schechtman, 2011). So I will not delve into this debate, even though it is doubtlessly relevant (cf. Bernecker and Michelian, 2017).

If there is something like the core self, then it is clear why authenticity should be considered a value or at least something to bear in mind. Choices that go against inclinations that logically and rationally follow from one's identity are a sort of “betrayal” of one's core self, to which one should rather be loyal—as this is, indeed, personal authenticity (e.g., President's Council on Bioethics, 2003). There are also social aspects that encourage a person to respect authenticity: the self we have manifested thus far generates expectations of consistency in those who interact with us. If we violate authenticity thus defined, we become unreliable (*qua* unpredictable) and risk getting away from reality, which has consequences over our lives.

In this respect, consider the example made by Glannon (2011) on memory editing. Imagine a scholar at the beginning of his career, who fails at his first major conference due to excessive nervousness: he might be so traumatized by this experience that he will be haunted by it at all future public speaking occasions. Therefore, he might resort to propranolol to weaken the emotional charge of the memory and start over without the weight of the past, so to speak. But if the next conference were also a failure, and the scholar resorted again to memory editing, this would lead to him not feeling anxious even though, in a way, he should, as his colleagues would still form a negative opinion of him. In other words, the scholar would risk being detached from reality, failing to understand his limits (which instead would be very clear to others). This could be seen as a case of non-authenticity, as the scholar would end up betraying the scientific standards he hoped to respect (as a full part of his core self), erasing his failures and not facing them (cf. Lavazza, 2016).

## Narrative identity and memory-modulation

Underlying the concepts of personal identity seen so far, there is the classic philosophical “question of characterization,” that is, the issue of “what makes a person the person that she is” (e.g., Kind, 2015). The concept of rigid identity can be seen as the extreme point of an ideal continuum, the other end of which can be the notion of extended identity. The latter seems to have a weaker normative value than rigid identity, and is supported by the current scientific data on the matter.

(Def4) *Extended identity* is based on the feeling of the bodily self, which is its core. The extended identity lies in interpersonal relationships, because it is not something original or innate, but something that emerges in the interactions of the individual (who has an innate instinctual endowment, which limits what can emerge from the interaction) and from social and cultural elements. The psychological dimension and the temporally distributed self, made of events and relationships, give rise to a more or less coherent narrative subject to rewriting (which, for some, does not reflect a self as a true entity).

This notion of identity is more consistent with the recent research in developmental psychology and psychology of personality, according to which consciousness is a purely relational concept (neither innate nor primordial) and emotions are first felt in the body and then internalized in the psychological world (cf. Neisser, 1995; Gergely and Watson, 1999; Habermas and de Silveira, 2008; Marraffa and Meini, 2016). In this vein, one may also consider Damasio's hypothesis (2010) that there is a hierarchy of selves, starting from the proto self (generated by homeostatic alterations of the body in front of environmental stimuli) to the autobiographical self.

The concept of extended identity can include both a naturalistic vision of the self and of identity, like Carruthers's, and a more psychologistic one, like that proposed by Schechtman. Carruthers (2011, 2015) does not commit himself to an ontology of the self and of identity, but argues in favor of an epistemology of the mind that in any case subverts the notions underlying the Cartesian notion of rigid identity. Without going into the detail of his complex view, it can be said that for Carruthers we can only know our thoughts (which are mainly opaque to us, as we cannot access them directly and introspectively) in the third person, thanks to the mindreading we also use with others. Indeed, first we access the mental states of others and then, thanks to this, we are able to use mindreading on ourselves. Our access to our mental states is interpretative:

“For present purposes, an *interpretative* process (…) is one that accesses information about the subject's current circumstances, or the subject's current or recent behavior, as well as any other information about the subject's current or recent mental life. For this is the sort of information that we must rely on when attributing mental states to other people” (Carruthers, 2009, p. 123).

Furthermore, Carruthers (2015) emphasizes the role of working memory and the neuronal activations that produce attention as opposed to the mentalistic conception based on beliefs, desires, objectives, and preferences. In other words, for him, when making our choices we do not have conscious access to a non-sensorial repertoire of contents (that is, the self), but we can only focus on what is present in our working memory.

In philosophical debates, the “question of characterization” about personal identity and the self has been mainly framed in the context of the so-called narrative identity. According to the latter, the characteristics and the events that make up an individual's identity are those that are connected in a more or less coherent way in a narrative structure. Here one can distinguish a line of philosophical reflection and a line of psychological investigation. I will first deal with the former and then with the latter.

In a useful example, Kind (2015, p. 127) notes that we can draw much information from the box score of a basketball game, including the final score and the players' performances. However, a reporter describing that same event may start her article from the last part of the match or even from many hours before the match (for example to say that one of the best players of a team was slightly injured in the warm-up). Therefore, the reporter will provide a narrative and not just information. For this reason, it may be particularly important to evaluate the use of memory-modulation interventions within the frame of narrative identity.

The idea of a narrative self and personal identity has been recently and persuasively defended by Schechtman (1996).

“The difference between persons and other individuals (…) lies in how they organize their experience, and hence their lives. At the core of this view is the assertion that individuals constitute themselves as persons by coming to think of themselves as persisting subjects who have had experience in the past and will continue to have experience in the future, taking certain experiences as theirs. Some, but not all, individuals weave stories of their lives, and it is their doing so which makes them persons” (Schechtman, 1996, p. 94).

In other words, an individual constitutes herself as a person by forming and operating with autobiographical narratives, which are shaped as the story of a person's life. The unity of a person is therefore the unity of an autobiographical narrative. The narratives are mainly implicit, have to be rather precise, can be accessed locally and need to have a correct relation to external facts.

But, in general, “facts about the literal identity of beings like us are inherently connected to practical considerations” (Schechtman, 2014, p. 10). And this practical unity revolves around the concept of *personal life*, mainly characterized by “the attributes of the individual—the physical and psychological capacities and internal structures that she possesses”; ”the kinds of activities and interactions that make up the individual's daily life“; and ”the social and cultural infrastructure of personhood—the set of practices and institutions that provides the backdrop within which the kinds of activities that make up the form of life of personhood become possible“ (Schechtman, 2014, p. 112–113).

Schechtman's notion of narrative identity can directly impact the evaluation of the processes of memory editing. In fact, Schechtman (2010) has made remarks on the Deep Brain Stimulation (DBS) used for Parkinson's that can be easily applied to interventions on memory. As known, neurosurgery can cause changes in the patient's character and inclinations. Schechtman believes that DBS can threaten personal identity because it can change—partly or fully—the subject's personality traits, aims and interests. According to her, if, after undergoing DBS, a patient shows a very different behavior, it can be said that she is, in a way, “a different person.” What matters is *how* the patient has changed: not as a result of what she has seen, learned or thought about, but through the direct effect of a passively undergone deep brain stimulation.

Schechtman sets two constraints to her idea of narrative identity. The first is the “reality constraint,” according to which the narrative of the self making up a person's identity should “fundamentally cohere with reality” (Schechtman, 1996, p. 119; a similar point to that made in Glannon's example). Obviously, a story may contain small factual errors or minor inconsistencies, but it cannot include clearly false claims or views of reality that are very different from those held by other people one interacts with. In this sense, it should not be possible to ”remove“ important memories, be it in a literal or metaphorical sense. De Grazia (2005) also supports a narrative theory of personal identity similar to Schechtman's, for which the narration must be made from the first-person standpoint but must also be realistic.

The second is the “articulation constraint,” according to which the self-narrative should be constructed by the subject in a way that justifies her choices and behavior, a person “should be able to explain why he does what he does, believes what he believes, and feels what he feels (Schechtman, 1996, p. 114); a similar point to that made in Erler's example). Schechtman claims that “the mechanism of personality change is important to its effect on forensic personhood and identity.” In fact, if a patient treated with DBS were to show a personality change, one would “have to acknowledge that his current passions and interests—the things he takes as reasons—were caused by manipulation of his brain.” As a consequence, this change would have to be considered “disruptive to his forensic personhood and identity in a way that natural personal development would not have been” (Schechtman, 2010).

It might be useful here to evaluate in terms of authenticity the alternative ways of modulating the mind/brain, that is, between classical psychotherapeutic techniques and chemical means. The memory-modulation techniques I am talking about seem to lack an active self-determination, as what matters is to achieve the goal (the removal of suffering) and reach success (see the examples made by Erler and Glannon). The subject's choice and personal path are less important, even if the subject consciously and voluntarily decides to take, for example, propranolol. In this case, we can say that the patient is passive, undergoing an external intervention, while in psychotherapy there is an active participation, an internal process that takes place over time. The two methods of intervention may have different degrees of effectiveness and sometimes drugs take less time. However, in psychotherapy there is a gradual change which one is aware of and agrees to one step at a time—one does not suddenly leap into another personal dimension.

Of course, this distinction may sometimes be less clear, because the therapist's guidance can lead the patient in directions toward which she would not otherwise go. In other words, the patient, who has to deal with traumatic memories, can find himself in a situation of emotional dependence (due to his situation), and cognitive dependence, on the therapist (due to the latter's expertise). In this sense, the patient's autonomy may be reduced, because he follows a path established by the therapist rather than his own. Unusual cases of unreal memories of abuse or multiple personality structures emerging during psychotherapy show the possibility of this risk (cf Hacking, 1995). However, pharmacological treatment and psychotherapy seem to still differ in terms of margins of autonomy of the self, because the conscious agency is less likely to evaluate and counter the ”directive“ effects of pharmacological treatment compared to psychotherapy, for the reasons set out above.

The individual should retain a certain ability to intervene to at least partially modify certain traits of his character that create discomfort or that he reflectively does not like, and this progressive construction should achieve a sense of unity: the various elements should be progressively integrated so that they can be subjectively recognized as one's own and the individual can actually identify himself in them. In other words, the pill treats the symptom, but does not build a character that will allow one to face other similar situations in the future. Furthermore, the ability to govern oneself is seen as a complex and stratified characteristic of personality (and as a value), which does not depend unequivocally on the biochemical balance of neurotransmitters.

However, it cannot be ignored in this regard that the most recent acquisitions of empirical psychology and cognitive science seem to point to a weak and fragmented ego, in which much of mental functioning and routine decisions take place within the cognitive unconscious. According to this perspective, there is no conscious active monitoring, if not when unexpected events happen, while environmental signals we are unaware of are continuously working to direct our behavior (e.g., Bargh, 2017). All this, as already seen, seems to contrast with the idea of authenticity as the reflection of a “rigid” ego, which remains largely stable over time.

In light of what has been said so far, the framework of narrative identity is particularly relevant because it is the main answer to the long-standing ”question of characterization.“ Within it, memory is crucial, because it allows us to construct the narrative according to the constraints of reality and articulation. Having truthful, reliable, and coherent memories (within the limits of a cerebral and psychological faculty of which we know all the “sins”; Schacter, 2003) causes our narrative identity to be functional to our subjective continuity. This way we can construct a self we can be conscious of and identify with. But respecting the constraints also means building a narration that is coherent with the environment and the social context in which we live, allowing us to have adequate interpersonal relationships. In fact, if we edit our memory at will, erasing for example unpleasant episodes, we could disregard our moral responsibility for some events or, say, the duty to remember a crime in order to testify against the culprit. Furthermore, weakening the emotional impact of a memory, which is crucial in remembering salient facts, can make the whole of our existential narrative less coherent, making us less able to explain some crucial developments of our life.

It has been claimed (Müller et al., 2017) that Schechtman's objection to DBS (and implicitly, therefore, to memory-modulation) is a case of naturalistic fallacy, entailing confusion between the property of being natural and that of being good. However, the matter seems to be more complicated than that and this, indeed, is why it is interesting to analyse memory editing procedures. In fact, Schechtman certainly wishes to preserve the narrative self and personal identity, but she doesn't present a naturalistic picture as opposed to a value judgment. The idea of “forensic personhood” has a normative aspect due to which some change processes are preferred to others. On the one hand, narrative identity is certainly extended identity, on the other it is also a prescriptive identity. In this sense, autobiographical memory is not a “natural” neutral element, to which one may associate a potentially negative judgment: it is part of the construction of the self and personal identity in a normative sense, and therefore has the same constraints.

In general, theories of narrative identity (cf. Lindemann, 2001) can be descriptive or normative. In the first case, the theories limit themselves to explaining how conceiving of one's life in terms of a narrative plays an important role in building one's personal identity, explaining the most relevant aspects of this way of constructing identity. In the second case, theories claim that we *should* conceive our life in terms of a narrative structure and that this can be relevant to achieve an ethically good life (cf. Kind, 2015). The main exponent of this normative approach is Taylor (1989), according to whom thinking of one's life as a coherent narrative is part of our attempt to reach goodness.

The development of narrative theories of identity has generally comprised an interweaving of descriptive and normative elements. In this sense, chemical interventions on memory are to be considered problematic precisely because they affect the truthfulness and coherence of one's existential narrative in a way that is not fully achieved by the subject, but thanks to an external intervention. A narrative deprived of relevant events is in fact equivalent to a false narrative—something that Schechtman and DeGrazia, among others, consider dysfunctional.

An influential line of psychological investigation is that of Jerome Bruner, who explicitly speaks of a narrative model of self-construction (Bruner, 1991, 1997, 2002). For Bruner there is no evident and essential self. We are the ones who continually construct and reconstruct a self according to the situations we find ourselves in, guided by the memories of the past and by the emotions and goals aimed at the future. The narrative mechanism, similar to that of literature, accumulates and stratifies stories, so that it does not start from scratch every time. But their link with the objective memory of events, according to Bruner, is quite weak. Rather, the narrative acts that construct the self are guided by the expectations of others and by implicit cultural models that suggest, if not impose, what the self should or should not be.

The creation-narration of the self, for Bruner, uses selective memory to adapt the past to the needs of the present and to the expectations of the future. Furthermore, it expands to adopt new beliefs and values, even though it maintains a degree of continuity over time despite the considerable changes it goes through. Identity can therefore be conceived as a verbalized meta-event which gives coherence and continuity to one's confused and chaotic experience. There is no “real” autobiography: ours is just one of the possible versions, a way of achieving coherence—a characteristic that both we and society tend to appreciate.

Bruner's essentially constructivist approach—that is, the idea that we create and recreate our identity through narrative—is not just a theoretical-normative view, but is based, in his opinion, on precise psychological mechanisms. Without the ability to tell stories we would not have an identity: this is shown by *dysnarrativia*, a neurological pathology, associated with syndromes such as Korsakoff or Alzheimer's, which involves serious damage to this capacity, canceling not only the memory of the past but the very sense of the self and of the other (Castelli et al., 2011; Baglio et al., 2012). The construction of the self therefore implies the precondition of good psychological and cerebral functioning. However, Bruner also insists on the cultural dimension of the construction of the self, which is not something innate, if not in its basic characteristics.

The narrative process of construction and reconstruction also involves a component of invention with respect to the past. As Bruner notes, this is due to both the normative component of the creation of identity, which often follows the indications of one's culture, and a naturalistic fact, that is, the fact that the human mind can never completely and faithfully recover what happened and was experienced in the past. In this vein, the unreliable and necessarily subjective character of memory can be combined with Bruner's constructivism to provide a narrative model in which the constraints posed by Schechtman have a lesser—or no—role, allowing one to say that pharmacological interventions on memory would not be a source of particular concern, especially if inserted into an appropriate narrative.

Contrary to narrative theories, the naturalistic idea of the self *a' la* Carruthers doesn't seem to incorporate explicitly normative elements, but rests on interpretation in the form of inference to the best explanation of the available empirical data (Chudnoff, 2016). A selective intervention on autobiographical memory only affects the integrity of the self insofar as a single memory is particularly relevant to the (largely subconscious and automatic) functioning of the mind. However, this is only the case with extreme PTSD patients, where a clinical intervention aimed at weakening the emotional salience of the given memory is the very condition for the person's recovery of autonomy. But this does not mean that there may not be a regulative component also in the naturalistic conception of the self.

## Memory-modulation and the battle of the self

The definition of rigid identity as such should imply an acceptance of memory modulation, as the very idea of a fixed and stable core entails that single memories cannot modify the self. However, the idea of rigid identity also entails the notion of authenticity, with its normativity. It implies the respect and acceptance of what nature and life have given every person (Sandel, 2009). The purpose is not to change an anthropological perspective that refers to an (implied) idea of the self as a real, self-conscious, free entity capable of managing its autobiographical memory as a storage of memories. The latter—positive and negative—are considered something given and valuable *qua* experience, which cannot be changed at will.

A very different view is offered by the psychoanalytic perspective, which is not part of the main focus of this article, but which deserves at least a mention at this point because it attributes a special role to memory with regard to self-development and self-transformation. Simply put, according to Freud, the patient's problems arise from secrets and memory lapses which concern the unconscious, by definition inaccessible directly. Free associations provide the analyst with the tools to unveil those secrets, to reconstruct those memories, and to reveal and modify the patient's internal resistance to knowledge and remembering. Treatment implies a definitive renunciation of the conflicting childish desires thus revealed (Mitchell and Black, 1995).

It can be affirmed that the purpose of this treatment is not to erase memories but to bring them to consciousness, in order to integrate them in the fabric of the other conscious psychic contents. In general, the memory of the traumatic experience is removed and delivered to oblivion, from where, however, it continues to act on the subject causing an uneasiness that she cannot deal with. The psychoanalytic perspective therefore does not seek the “dampening” memories—something that it considers impossible—but rather the reestablishment of associative links and the reintegration in the self of something that has been removed. The therapeutic act lies in the ability to narrate the self in a coherent and understandable way. It is a matter of reconstructing the continuity of one's representations of the self and of the world, which were interrupted by an event to which one has not been able to give meaning within one's life story.

Winnicott's idea of True and False Self goes in the same direction of memory recovery as an act of disclosure of the False Self, while erasing dysfunctional memories or a mitigation of their emotional capacity would probably end up preventing a recovery of the True Self. Indeed, when the infant lacks a good enough parenting, she takes shelter in fiction and, through the False Self, she “builds up a false set of relationships, and […] even attains a show of being real” (Winnicott, 1960). But the False Self is a defensive structure that is cloaked with objectivity and elements of the external world to protect the more fragile True Self, in order to protect the individual from being crushed by emptiness and inauthenticity.

However, in Western culture there has been a passage from a psychological view of the human being to a view that progressively relates to the body, and to the brain in particular (Rose, 2007 ch. 7). Ultimately, the idea is that we are biochemical selves, in which the functional (mental) elements converge into the cerebral aspects. The truth about our Self, provided it exists, comes from scientific research with its experimental models and diagnoses that attempt to categorize it objectively. And science only works with the brain and its potential modifications, the implementation of which requires drugs that are molecules as much as those contained in the brain itself. According to Rose, if the mind is just brain activity and psychic disorders are biochemical imbalances, then we are faced with a new ontology that inevitably bears more general consequences.

This point is very important. That psychiatric diagnosis is now done at a molecular level, by recognizing an ever closer link between neurochemistry and behavior can only be considered a form of progress, as better knowledge allows for the cure of disorders that were previously untreatable. Too low or too high levels of a given neurotransmitter as well as inadequate neuronal transmission mechanisms cause variations from a “normal” or “functional” to a “dysfunctional” state for the subject. The scientific idea that now subjects can be treated chemically also contributes to the creation of a cultural metaphor.

For example, Rose writes, Prozac is a drug that only affects serotonin reuptake, selectively acting on the subject's mood. On the one hand, it does not have any significant side effects, but on the other hand, it promotes the view that there are single isolated systems producing identifiable diseases that are not associated with other causes. The disorder is only organic, society does not matter, nor do personal relationships and social interactions. There is only one clear target, which is within us. We are made of many small pieces that can mostly be cured.

In general, says Rose (2007), it appears that new psychiatric drugs cure somewhat vaguely defined diseases, whose very existence is sometimes questioned. They do not cure a specific pathology—in the classic sense—but modify the ways in which salient events (emotionally or objectively significant) of life are experienced and understood by people. These drugs appear to be targeted at the so-called “biovalue,” that is, the conception of what human beings are or should be, which is internalized in the idea underlying these drugs as norms, values, and opinions. There is an ethics inscribed in the molecular composition of these drugs: they carry and stimulate particular forms of life in which the “true self” is both “natural” and to be constructed. These drugs have important effects not only in terms of how they treat patients but also because they affect the way in which we see, interpret and describe ourselves and the world.

If autobiographical memory can be modulated at will, without getting to ”cosmetic neurology“ (Chatterjee, 2004), then what follows is the idea that the individual can be adjusted to be more ”functional“ (to society, to consumerism, to production etc.). This is not, of course, a direct and enforced form of control. However, the choice to take psychotropic drugs (including propranolol, in the future) may be due to a strong social pressure or a cultural climate, also because the mindset promoted is that the cure will restore the person's true self. The neurochemical coordinates of a “normal” or ”efficient“ self are in fact established by science considering the average brain physiology, regardless of all other variables. Once the neurochemical self is identified, it needs to be restored whenever it loses its balance. But whether this self is desirable is open to discussion.

Nevertheless, the consideration above should not lead to an anti-psychiatric stance that completely discards science and its findings. Consider the mentioned topic of dysfunctional memories: on the one hand, they can lead to the stigma of mental illness in relation to PTSD; on the other hand, they can create pressure for treatment by means of memory-modulation, with a change of autobiographical memory. Modulating a painful autobiographical memory (or canceling it altogether, which may be possible in the future) has undeniable personal and social consequences that go beyond the objective clinical (and mostly beneficial) intervention on the disorder identified as the hyperactivation of the circuits described in paragraph 2. The decision to intervene on one's autobiographical memory—and potentially on one's self—also implies value decisions involving the subject's autonomy and identity, as well as implicit social values (materialism, efficiency, and scientific humanitarianism being in favor of the cure; classical humanistic and religious values being against it).

One should also consider Michel Foucault's remark that medicalization—linked to the naturalization of the biochemical self—implies a passage from the legal regulation of society to its normalization, with the application on the social body of a set of knowledge, institutions, and controls that structure the life of the population according to binary criteria (normal-pathological, legal-devious, healthy-sick). Medicalization can take on a disciplinary function: it can structure, and control individual and collective physiology in order to qualify it normatively in educational, productive, and consumerist institutions (cf. Pandolfi, 2006).

In general, it is interesting to note that, according to Foucault, self-care and ethics as practices concerning the most important aspects of freedom have become the most advanced fronts of political struggle in our societies, where those in power try to limit and influence the choices of everyone else. Self-care and ethics bring into play the idea of human nature as the target of all manifestations of social power. Human practice is losing its traditional center identified with the self, consciousness, or other aspects of personal identity. The Multiple Personality Disorder/Dissociative Identity Disorder (MPD/DID) debate is linked to this scenario and involves what Hacking (1995) refers to as politics of memory. In his view, the new memory sciences, developed since the late nineteenth century, have taken the soul away from religion and handed it over to science. In this way, “moral struggles” have become objective and impersonal. For example, as suggested (albeit in a different sense) by Herman (1992), the new knowledge and theories about memory functioning have contributed to the development of feminism, a political movement that also exploits traumas connected to MPD as a tool to show the subordinate condition of women and pursue their emancipation.

According to Braude (1991) multiple personalities give rise to distinct autobiographical memories which, however, are not indexical, being described in the third person. They would, therefore, be based on a unifying self or mind: “It seems compelling to appeal to an underlying synthesizing subject who simply evolves into a multiple as a complex and creative response to various life situations” (Braude, 1991, p. 173). In fact, the primary awareness seems to be continuous and unified also in subjects diagnosed with MPD, which therefore could be an extreme dissociative disorder. But what's at stake here, regardless of the several different diagnoses and scholarly opinions on the phenomenon, is above all the subjectivity of the descriptions, both on the part of the patient and on the part of the therapist. Therefore, the “battle of self” linked to autobiographical memory can be traced back to purely normative models, because there is no scientific consensus either on the disorder or on the way in which the traumatic memory should be treated.

In accord to Foucault, even the organic unity of the body tends to fragment itself, so as to be reduced to its genetic bases. And if genetic foundations can be changed, natural inequalities—once considered irremediable—can be easily modified. In a sense, there is nothing natural left and this may mean that there is no longer a structured and precise self that stays stable over time, since everything becomes fluid and malleable at will. Genetic susceptibility to PTSD could thus be treated in ways similar to memory modulation, with a preemptive intervention that would prevent negative and painful memories from causing discomfort to the individual. The underlying idea is that of self-construction, by which the self is an open field for experimentation. Think of the attempt to contain the response of the immune system after a transplant, a response that is completely natural and adaptive, but has to be countered to allow the organism to welcome a new part. Something similar can happen with memory: the chemical modulation of memory goes against its biological functioning, which tends to emphasize memories that may be important to our survival in a certain environment, although they may be painful for the individual experiencing them.

Autonomy, a fundamental value of modernity that implies freedom of choice and ability to act independently of others, may thus give way to a new normativity: that of “appropriated affirmation” (Ehrenberg, 2010). Now that social bonds are weaker, people must be able to rely on their interiority and subjectivity, which must be functional. However, the traditional ban against being oneself now turns into the “obligation” to become oneself—and it remains to be seen what “self” one should become. So, biopolitical battles are now fought on an unsteady ground, with no invariants or hard cores, but only varying and modifiable physical and social conditions, up to the post-anthropological and post-humanist hypothesis of machine-body hybrids, with embedded or uploaded external memories constructing a blurred self.

## Conclusion

It is unquestionable that, over time, people change the way they react to stimuli and events (and these reactions can be objectively assessed, contrary to the more “fleeting” notion of the self). It is equally unquestionable that autobiographical memory is an important component in relation to the subject's response, as it contributes to building the subject's repertoire of reactions and affects the probability of such responses. Change as such does not imply a change of identity or self. Unless one adopts a deconstructionist view of the self (Metzinger, 2009; Strawson, 2009), personal identity and the self are usually taken to be precisely what persists in a person through change.

However, the possibility of memory editing (which, for now, is still fairly limited) entails consequences on the self, broadly understood as the subject responsible for choices and behaviors (while taking into account the differences related to the various notions of self on the market). Objections to the modulation of autobiographical memory are mainly linked to regulative conceptions of the self, related to descriptive conceptions of the self (like the “narrative” one).

One of the ideas underlying such objections is that “natural” change in one's self is gradual, allowing one to foresee how one may be *after* as opposed to *before*. Instead, the choice of drug-induced oblivion implies an immediate transition with no intermediate stages, making such a comparison impossible. Another view against memory-editing interventions is that change should be *purposeful*, that is, it should aim at liberating the “true” self, developing elements that were already present in the self since childhood. In this sense, experimental philosophy has recently shown that people tend to describe positive changes (for example, acquiring qualities generally seen as desirable, such as self-control vs. impulsivity) as consistent with their identity, while judging negative ones (for example, being violent) as a deviation from their true self (Tobia, 2015).

This intuition, despite being often fallacious *qua* based on a prejudice, can lead to an important consideration. Indeed, it seems that personality changes differ based on the subject's ability to account for them. If one can explain how and why one has changed (albeit with the limitations of a subjective explanation), it means that probably this change is at least partly consistent with the self as it was *before* the change. And yet this criterion seems hardly objective. In fact, as we have seen, the ideas of naturalness and authenticity stem from specific conceptions of the self and its relation to autobiographical memory—in particular from an idea of “rigid identity,” as I have called it. This conception of the self goes against many elements of recent empirical research, whose results are more aligned with what is termed “extended identity.”

Constructivist conceptions do not regard the self as original and complete entity but as emergent, fragmented and only *narratively reconstructed* as a whole. For these conceptions, the single (non-systematic) modulation of autobiographical memory is not a relevant problem, so long as it does not imply detachment from reality, understood as the set of material and social conditions in which the person can flourish and from which she gets feedback on her actions.

There also are conceptions that are predominantly normative, based on an extremization of the available scientific data. In this perspective, a so-called “neurochemical self” is the battlefield of external influences, and memory modulation can be considered an invasive tool, extrinsic to the process of personal identity construction. In this sense, one can speak of self-depletion, as the self is manipulated and impoverished—for example—due to an efficientist social pressure relying on a purely brain-based conception of identity.

On the other hand, a conscious choice to modify one's memory to modulate painful and paralyzing memories, within constructivist self-conceptions, appears as an effective tool that, however, is not qualitatively different from other narrative strategies to harmonize one's identity and make it functional in specific environmental conditions. In this sense, one can talk about self-improvement, in a perspective from which memory-editing is not akin to cosmetic neurology but rather to self-care and self-enhancement.

But, as we have seen, in the general perspective of narrative identity, both on a descriptive level and above all on a normative level, memory-modulation interventions are problematic because they violate the constraints of a functional and coherent narrative. Instead, models that adopt a more openly naturalistic perspective seem to pose fewer normative restrictions to the partial modification of one's autobiographical memory, as these modulations do not seem to concern the functioning and balance of the autobiographical self-memory system.

It must be repeated here that forgetting traumatic experiences or generically unpleasant ones represent different sides of the wider problem of forgetting. Although it is difficult to draw a clear distinction, as such situations should always be assessed individually, there are two extreme situations that are more easily classified along the continuum of possible interventions on the “self and autobiographical memory” system. On the one hand, clinical situations related to serious trauma; on the other hand, pure cosmetic neurology. The first ones are those that arouse the least concern with respect to their manipulation, while the latter are those that arouse the highest. However, the former also present relevant issues, as in the hypothetical case of witnesses of the Shoah (cf. Lavazza and Inglese, 2013).

The present discussion, which is undoubtedly partial, has shown that it is difficult to break the strong bond between descriptive and normative conceptions on the self when it comes to the potential modulation of autobiographical memory. But this twine will be more easily “undone” at an analytical level when neuroscience and psychological research achieve a clearer understanding of the mechanisms that make up what we call self and personal identity.

## Author contributions

The author confirms being the sole contributor of this work and approved it for publication.

### Conflict of interest statement

The author declares that the research was conducted in the absence of any commercial or financial relationships that could be construed as a potential conflict of interest.

## Footnotes

^1^ Animalism is one of the theories that, like Lockeanism, seek to explain what human beings are. According to it, human beings are biological organisms, that is, human (i.e., thinking) animals. Animalism falls under the physical approach to personal identity (together with bodily theory and brain theory), and argues that our identity consists in being the same biological organism over time. This theory seems to give less importance to memory in relation to personal identity, and I will not deal with it here.
